# Preparation and characterization of self-cured geopolymer binder using metakaolin precursor

**DOI:** 10.1038/s41598-025-32571-0

**Published:** 2026-01-24

**Authors:** H. M. Khater, S. A. ElMoied

**Affiliations:** Housing and Building National Research Center, 87 El-Tahrir St., Dokki, P.O. Box 11511, Cairo, Giza Egypt

**Keywords:** Metakalloin, Portland cement, Activation, Self-cured, Engineering, Environmental sciences, Materials science

## Abstract

This study presents an advancement in “Self-cured geopolymer” technology, aiming to enable ambient-temperature curing. The experimental work systematically investigated two primary strategies: the incorporation of Ordinary Portland Cement (OPC) as an additive and the modification of manufacturing processes. The results demonstrate that the supplementary calcium from OPC enhances the geopolymer’s curing regime, yielding superior early-age strength and mechanical properties. Notably, the latent heat released from the reactions of high-energy compounds (e.g., OPC and activators) was found to be a significant internal heat source, functionally comparable to external heat curing. The synergy of these approaches establishes a feasible pathway for developing “Self-cured geopolymer cement” that achieves substantial mechanical strength under ambient conditions. The developed Self-cured geopolymer techniques, there are potentials that could increase the commercial viability of geopolymers as a construction material in construction industry by eliminating heating process and preparation of alkaline liquids as well as it could make a solid contribution to the field of low-carbon binder development. Potential application of Geopolymer cement powder as conventional OPC by just adding water. The results of the current work showed that the strength values reached abobit 45 MPa for 20% replacement (optimum dose) after 28 days of curing, while for one-part geopolymer mix reached to 48 MPa for 40% replacement (optimum dose) after 28 days curing,

## Introduction

Ordinary Portland Cement (OPC) continues to dominate the construction industry due to its cost-effectiveness, high mechanical strength, and proven long-term durability, making it widely used in infrastructure, roadworks, and specialized structural applications^1–5^. However, OPC production contributes approximately 5% of global CO₂ emissions, which has sparked growing efforts to develop more sustainable, low-carbon binder systems^6^. The transition to these alternatives is not trivial: any replacement must not only reduce environmental impact, but also achieve the high performance, durability, and workability required for widespread use^7–10^.

One promising path is through low-carbon binders, particularly geopolymers derived from industrial by-products such as fly ash, slag, and calcined clays like metakaolin^11,12^. Geopolymers form three-dimensional aluminosilicate networks via the alkali activation of these precursors, and, compared to OPC, they avoid the high-temperature calcination step, thereby substantially reducing CO_2_ emissions. Geopolymer concrete (GPC) is known for its high early-age strength, low permeability, and excellent resistance to chemical attack, attributes that derive from its unique gel chemistry, precursor composition, activator type and concentration, and curing regime^13,14^.

Advanced geopolymer systems often utilize one-part (dry-mix, “just-add-water”) or two-part (liquid activator) designs, each offering different benefits in terms of handling and reactivity^15^. Given increasing regulatory and sustainability pressures, geopolymer technology is being positioned as a key solution for green construction^16^. Geopolymers stand out as promising binding materials because of their substantially lower carbon footprint compared to traditional cement. Their production process is less energy-intensive and produces fewer CO_2_ emissions since it does not require calcination, leading to more environmentally friendly concrete. This reaction forms an amorphous or semi-crystalline structure via the interaction between aluminosilicate-based precursors and alkaline activators.

While typical geopolymer precursors like fly ash, slag, and metakaolin^17^ yield concrete with superior early strength, low permeability, and chemical resistance, the one-part “just add water” system remains under-explored despite its potential^18,19^. Its geopolymerization involves ion exchange, hydrolysis, and network breakdown^20,21^, but unlike OPC, its reaction mechanisms are highly dependent on the precursor^22,23^. Synthesis methods like the direct method and alkali fusion activation^24,25^ are used, with mechanical performance heavily influenced by the alkaline activator. Anhydrous sodium silicate is the predominant solid activator due to its high efficiency with aluminosilicate materials^26,27^. An emerging strategy to further enhance geopolymer performance is the incorporation of magnesium-aluminate (Mg–Al) nano-spinel (MgAl2O3). Recent work by Mohsen et al.^28^ demonstrated that adding nano-spinels to alkali-activated slag significantly refines the phase composition, reduces meso-porosity, and increases compressive strength compared to control samples. The nano-spinel acts as a nucleating agent, promoting the formation of denser gel networks and more crystalline or semi-crystalline phases, which ultimately strengthens the geopolymeric matrix and improves dimensional stability^29^.

In parallel, valorizing toxic sludge—such as industrial sludge or water-treatment residues—in geopolymer formulations is gaining traction. The use of sludge not only addresses waste management challenges, but also provides silica, alumina, and other reactive species for geopolymerization. However, the presence of organic matter, variable chemistry, and heavy metals in sludge can complicate geopolymerization and pose leaching risks.

This is where hydrothermal treatment (HT) plays a transformative role. Hydrothermal curing—typically involving exposure of geopolymer pastes to elevated temperature, pressure, or steam—has been shown to significantly accelerate geopolymerization, resulting in improved early-age and long-term compressive strength. It also promotes the conversion of amorphous gels into more crystalline or semi-crystalline phases, thereby reducing the amount of unreacted material and contributing to a more refined microstructure. In addition, HT enhances the immobilization of heavy metals by densifying the matrix, lowering overall porosity, and facilitating the incorporation of toxic ions into more stable binding phases. As a result, the overall durability of the geopolymer is improved due to the formation of a more cohesive and tightly interconnected binder network.

For example, Ramadan et al.^30^ reported that hydrothermal treatment (6 bar, 6 h) significantly improved mechanical efficiency and immobilization behavior in sludge-modified geopolymeric systems. Moreover, life-cycle assessments and valorization studies have shown that inorganic residues derived from hydrothermal-treated sewage sludge can be successfully incorporated into geopolymer binders without unacceptable leaching, while contributing to the mechanical performance of the final material^31^.

Mechanistically, the synergy between Mg–Al nano-spinel and hydrothermal curing in sludge-based geopolymers can be very powerful: the nano-spinels act as nucleation centers under hydrothermal conditions, facilitating rapid phase development and crystallization, while HT densifies the structure, reduces porosity, and stabilizes heavy metals within the network^32^. This synergistic effect can lead to a geopolymer that is not only high-strength and durable, but also environmentally safer by immobilizing toxic species in a stable matrix.

Table 1 represents the summary of previous studies on metakaolin- and fly ash–based geopolymer systems, including one-part solid activators, hybrid binders, and performance modifiers.

In this context, the present research aims to develop self-cured geopolymer pastes by partially replacing metakaolin with up to 30 wt % OPC, and to formulate a one-part “just-add-water” geopolymer paste containing up to 50 wt % OPC. We will incorporate Mg–Al nano-spinels to improve phase development and strength, and we will incorporate toxic sludge to valorize waste and assess immobilization. Crucially, we will apply hydrothermal curing regimes to exploit synergetic benefits: accelerating geopolymerization, refining phase composition, reducing porosity, and locking in hazardous ions. The resulting materials will be characterized by FTIR, XRD, SEM, and porosity measurements, and their compressive strength and leaching behavior will be evaluated comprehensively. By correlating microstructural modifications with mechanical properties and environmental performance, this study seeks to develop optimized, sustainable, high-performance geopolymer formulations that address both construction and waste management challenges.

## Materials and methods of investigations

### Materials

**Fig. 1:**
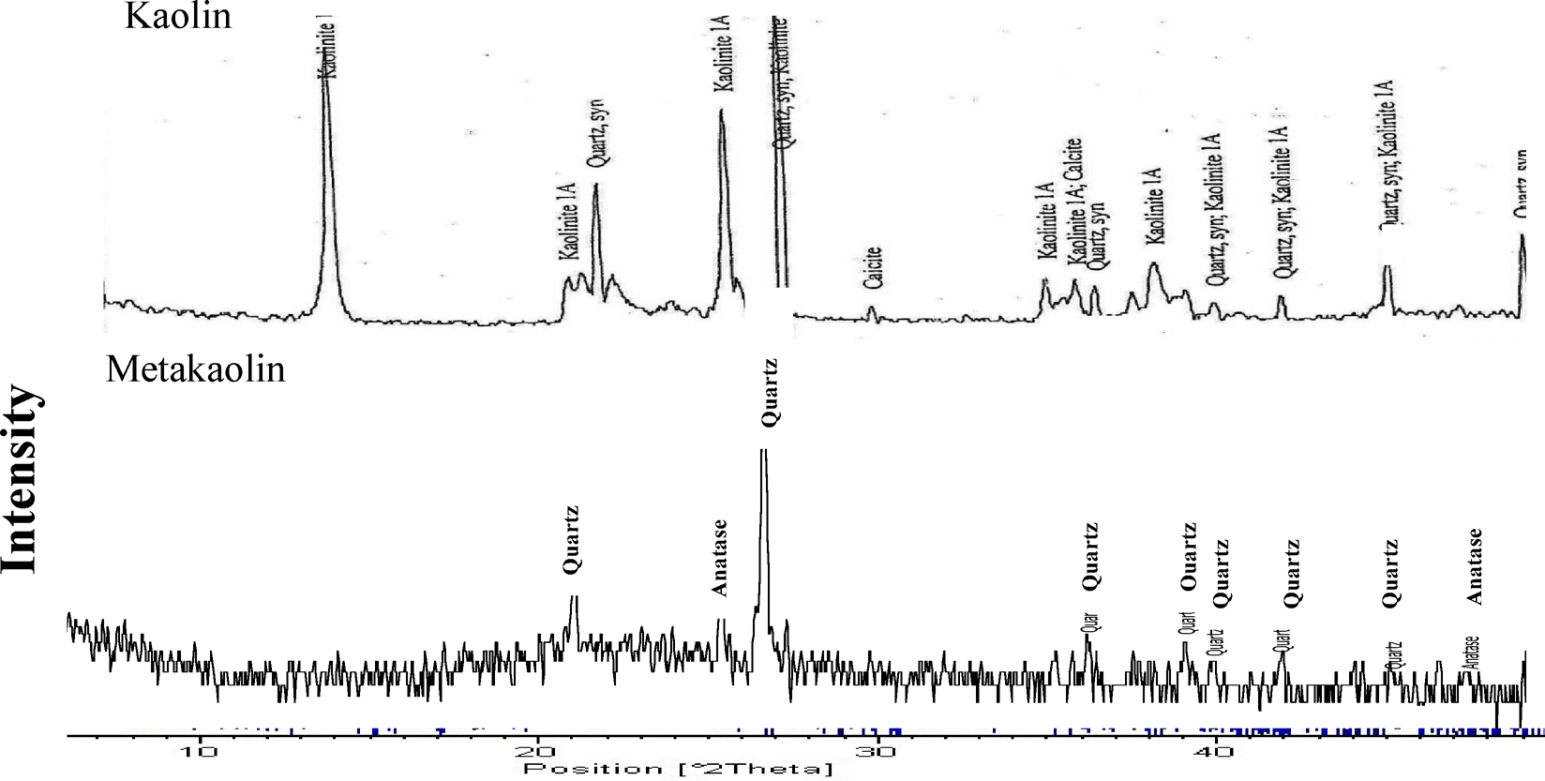
X-Ray diffraction pattern of the starting materials.

Powdered kaolin, sourced from the Sinai Governorate in Egypt, was used as the primary raw material in this study. Figures 1 illustrates that its main crystalline phases are kaolinite and quartz. Metakaolin (MK) was produced by calcining the kaolin at 850 °C for 2 h, transforming the kaolinite while quartz remained the dominant crystalline phase in the MK. The resulting MK powder exhibited a specific gravity of 2.51 and a fineness of 370 m²/kg whereas their chemical composition tabulated in Table 2.

**Fig. 2:**
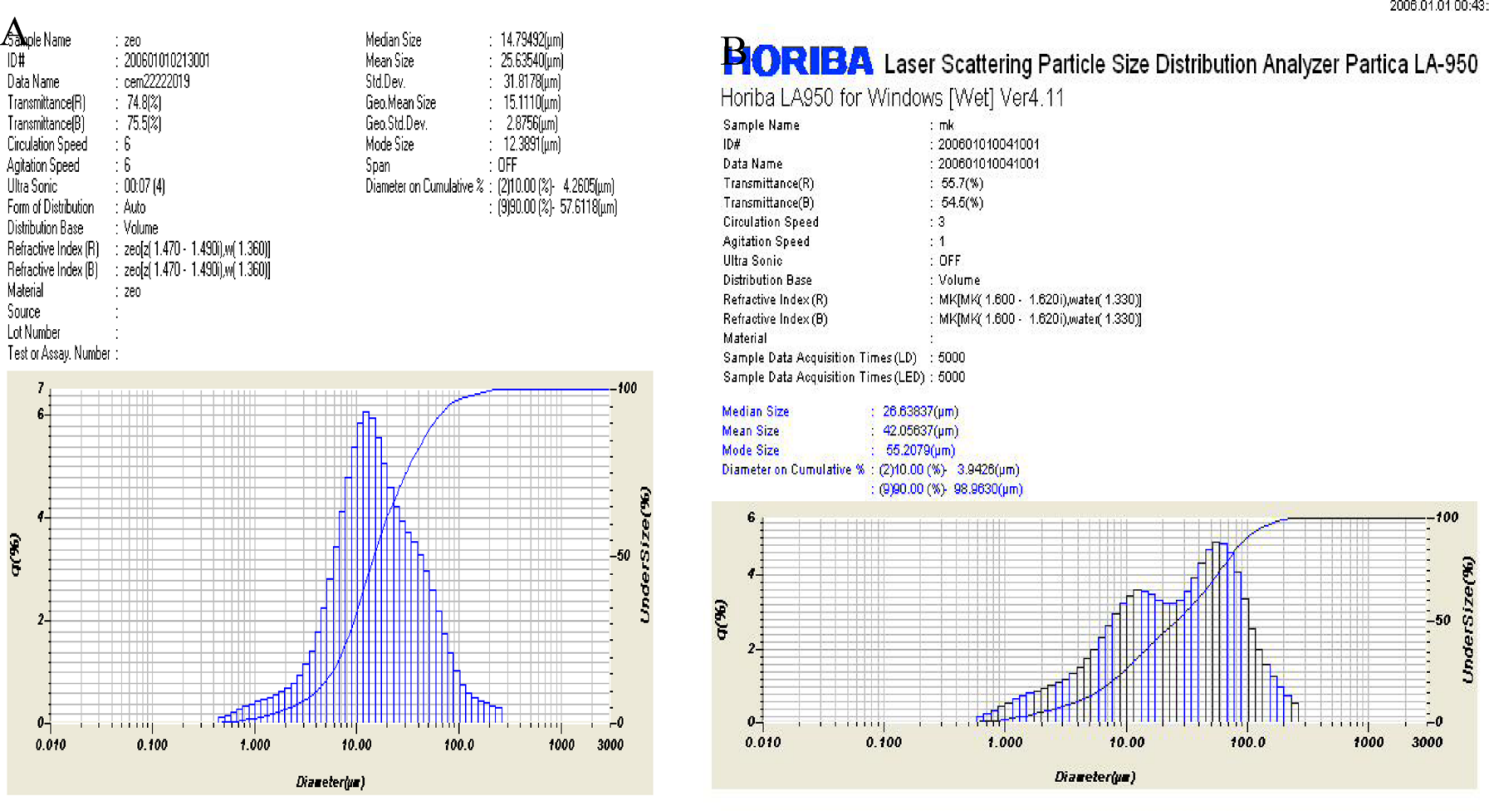
Laser particle size distribution of **A** Portland cement, **B** metakaolin.

Figure 2 depicts the laser particle size distribution of both prepared metakolin as well as Portland cement, where metkaolin was of mean size 42.056 μm, while Portland cement was of mean size of 26.635 μm.

### Mixing and curing regime

The current study investigates two mechanism for geopolymer prepaption. The first type involves a traditional cement–geopolymer paste, in which metakaolin (MK) is partially replaced with Ordinary Portland Cement (OPC) at incremental levels of 0, 5, 10, 15, 20, 25, and 30%. To activate the mix, an alkaline solution comprising 10 M sodium hydroxide^47^ (in a one-part ratio) and sodium silicate (in a two-part ratio) was used. Notably, the sodium hydroxide solution was prepared one day in advance by dissolving NaOH pellets in water.

In preparing this type of geopolymer, the dry materials (MK and OPC) passed 90 μm first thoroughly mixed for 5 min to ensure uniformity. Subsequently, the alkaline solution was gradually added until a workable paste was obtained. The resulting paste was then poured into molds that had been lubricated beforehand. Finally, the molds were covered with plastic sheets in order to minimize water evaporation during the setting process. The detailed mix proportions are presented later in Table 3.

The mixture for one –part regigme incorporate the specified activator dosage and about 10% fresh water (Table 3), followed by drying at 80◦C for 24 h to form the one-part geopolymer composite. Following the 24-hour period, the composite was ground to achieve a particle size of less than 90 μm, shaped into molds, and left undisturbed at room temperature for 24 h within cubic molds. It was then exposed to a curing temperature of 40 ◦C with (100%) relative humidity (R.H.) to induce a reaction under moderate temperatures, simulating global temperature extremes. The specimens underwent compressive strength tests after being removed from the curing environment and completely dried at 80◦C for a full day. After being crushed, the specimens were kept in an airtight container until they could be examined. They were then preserved in a methyl alcohol/acetone solution, to stopping hydration.

### Setting time of self-cured geopolymer pastes

Table 4 shows that increasing OPC content (0–30 wt% replacement of metakaolin) dramatically accelerates the setting of self-cured geopolymer pastes: the control mix without OPC exhibits an initial setting time of about 410 min and a final setting time of 430 min, whereas with progressive OPC additions these times shrink to as little as 8 min (initial) and 20 min (final) for 30 wt% OPC. This acceleration can be explained by multiple interacting mechanisms documented in the literature. First, the exothermic heat from cement hydration raises the local temperature, promoting faster dissolution of aluminosilicate species and speeding up geopolymerization; similar thermal acceleration phenomena have been observed in geopolymers containing calcium sources^48^. Second, hydration products such as C–S–H (or C–A–S–H) act as heterogeneous nucleation sites, thereby facilitating rapid precipitation of hybrid gels and reducing the induction period. Evidence for early precipitation of C–S–H in calcium-modified metakaolin geopolymers has been shown via XRD analysis^49^. Third, dissolved Ca²⁺ from OPC modifies the pore-solution chemistry, enabling the formation of calcium-containing aluminosilicate phases (e.g., hybrid C–A–S–H/N–A–S–H) that tend to rigidify faster than pure sodium-aluminosilicate networks; this has been supported by microstructural studies showing co-existence of C-S-H and N-A-S-H gels^50^. Fourth, early cement hydration consumes water and produces solid hydration products, which reduces the free water fraction in the paste and densifies the microstructure so that stiffening occurs more quickly in line with findings that adding calcium significantly reduces setting time^51^.

These trends (faster setting with Ca-rich addition) accord with previous studies of hybrid OPC–geopolymer systems; in particular, the effect of cement hydration heat and hybrid gel formation on both early kinetics and mechanical development has been well documented^21,23^. Methodologically, robustness of such accelerated setting systems should be backed by replicate measurements, internal temperature monitoring, and microstructural characterization (e.g., XRD, FTIR) to confirm the formation of hybrid phases. Practically, the rapid setting afforded by higher OPC contents can be exploited for fast-repair or rapid-turnaround applications, but workability becomes a challenge; strategies such as alkali-stable retarders or staged mixing (or controlling OPC ratio) may help manage this tradeoff.

Table 4 illustrates the setting time values for self-cured geopolymer mixes having various content of portlland cement up to 30%. The results depicts the gradual decrease of both intital and final setting time for all mixes with increasing cement content as the hydration process in cement matrix leads to steam activation for geopolymerization reaction leading to the decrease in the setting time reaching to 8 min intial setting up on using 30% replacement as compared with 6 h and 50 min for control mix that haven’t any cement content.

### Methods of investigation

The oxide composition of the raw materials was determined using X-ray fluorescence (WD-XRF) on an Axios Sequential Spectrometer (Panalytical, 2009). Compressive strength was measured with a German Brüf pressing machine in accordance with ASTM C109^52^. Mineralogical analysis was conducted via X-ray diffraction (XRD) using a Philips PW3050/60 Diffractometer, while the amorphous structure was characterized by Fourier transform infrared (FT-IR) spectroscopy^53,54^. The morphology and microstructure of the hardened composites were examined using scanning electron microscopy (SEM) equipped with energy dispersive X-ray spectroscopy (EDX). Prior to SEM imaging, the samples were sputter-coated with gold.

## Results and discussion

### FTIR

**Fig. 3:**
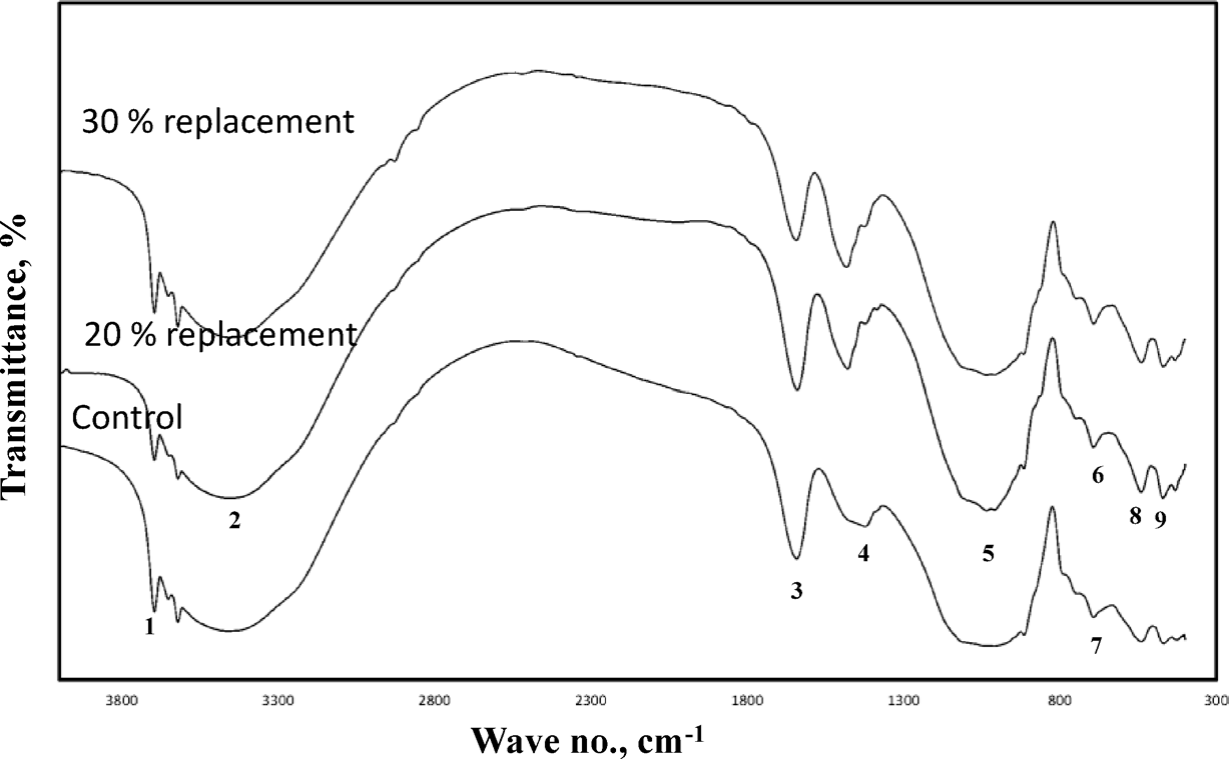
FT-IR spectra of 90 days self-cured geopolymer paste having various ratio of Portland cement [1: Stretching vibration for O–H for Portlandite, 2: Stretching vibration of O–H bond, 3: Bending vibration of (HOH), 4: Stretching vibration of CO_2_, 5: Asymmetric stretching vibration for ettringite, 6: Asymmetric stretching vibration (Si–O) for CSH,7: Out of plane bending vibration of CO_2_, 8: Out of plane vibration of (Si–O–), 9: In plane vibration of (Si–O–)].

As shown in Fig. 3, the intensity of the asymmetric T-O-Si stretching band at approximately 997 cm⁻¹, a representative band for geopolymer formation^55–60^ increases in the 28-day metakaolin geopolymer pastes as the Portland cement content is raised, reaching a maximum at a 20% replacement level.

This is accompanied by gradual decrease in the intensity of asymmetric band of Si-O-Si for non-solubilized silica at about 1100 cm^− 1^, which results in an enhancement in the geopolymerization reaction that reflected positively on the intensity of the aforementioned band. On the other hand further increase in the Portland cement content to 30% results in partial reduction in the intensity of the asymmetric band as a results of dilution effect by added cement leading to disturbance in the geopolymeriztion reaction and the increased tendency to form CSH rather than NASH gel.

This interpretation is supported by the Portlandite (CH) band at ~ 3520 cm⁻¹. Its intensity decreased with a 20% cement incorporation, indicating enhanced geopolymerization. However, at 30% cement, the CH band intensified due to the propagation of the hydration reaction, which liberates Portlandite as a by-product. The initial increase in geopolymer band intensity is attributed to the continuous dissolution of aluminosilicate sources, driven by self-curing from the internally generated heat. This intrinsic heat, comparable to external thermal curing, enhances the reaction regime, forming amorphous geopolymer constituents and widening the asymmetric band at ~ 997 cm⁻¹.

**Fig. 4:**
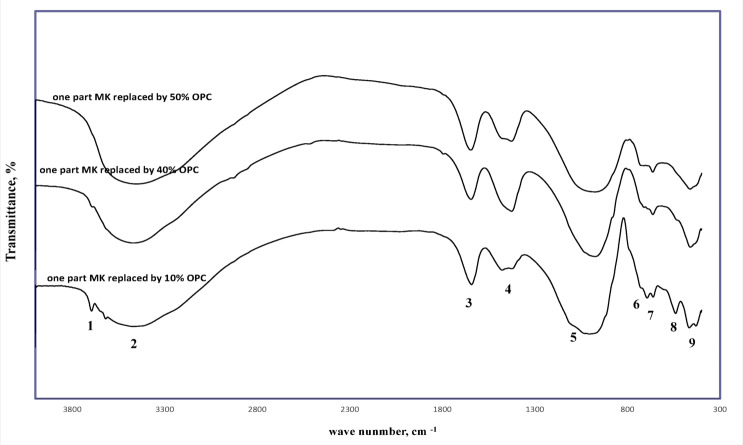
FT-IR spectra 90 days one-part self-cured geopolymer paste having various ratio of Portland cement. [1. Stretching vibration for O–H for Portlandite, 2: Stretching vibration of O–H bond, 3: Bending vibration of (HOH), 4: Stretching vibration of CO_2_, 5: Asymmetric stretching vibration for ettringite, 6: Asymmetric stretching vibration (Si–O) for CSH, 7: Out of plane bending vibration of CO_2_, 8: Out of plane vibration of (Si–O–), 9: In plane vibration of (Si–O–)]

In the one-part mix (Fig. 4), the dry mixing process itself generated significant heat, further aiding the curing. The added calcium in the GeoPC system improved early strength via the precipitation of C-(N)-A-S-H gels and provided latent heat from the reaction of high-energy compounds^61^. This is evidenced by a shift of the asymmetric band to ~ 950 cm⁻¹ and the complete disappearance of the Portlandite band at 40% cement. A further increase in cement content broadened this band and reduced its intensity, likely because the hydration reaction consumed dissolved silica to form C-A-S-H/C-S-H gels instead of N-A-S-H gel.

### XRD analysis

**Fig. 5:**
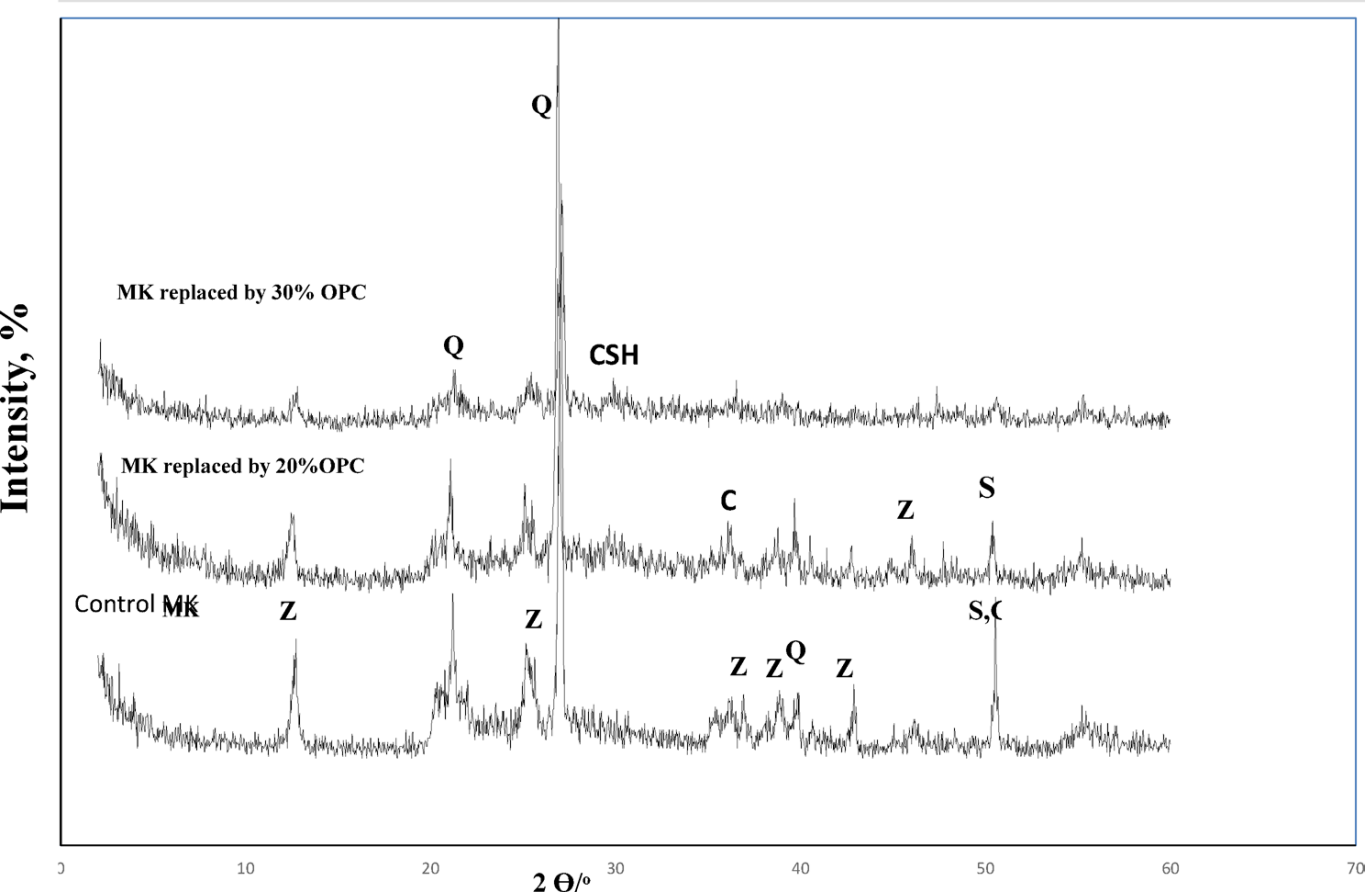
XRD pattern of 90 days MK-geopolymer paste having various Portland cement ratio [*Q* Quartz, *Z* Zeolite, *S* Sodalite, *CSH* calcium silicate hydrate].

**Fig. 6:**
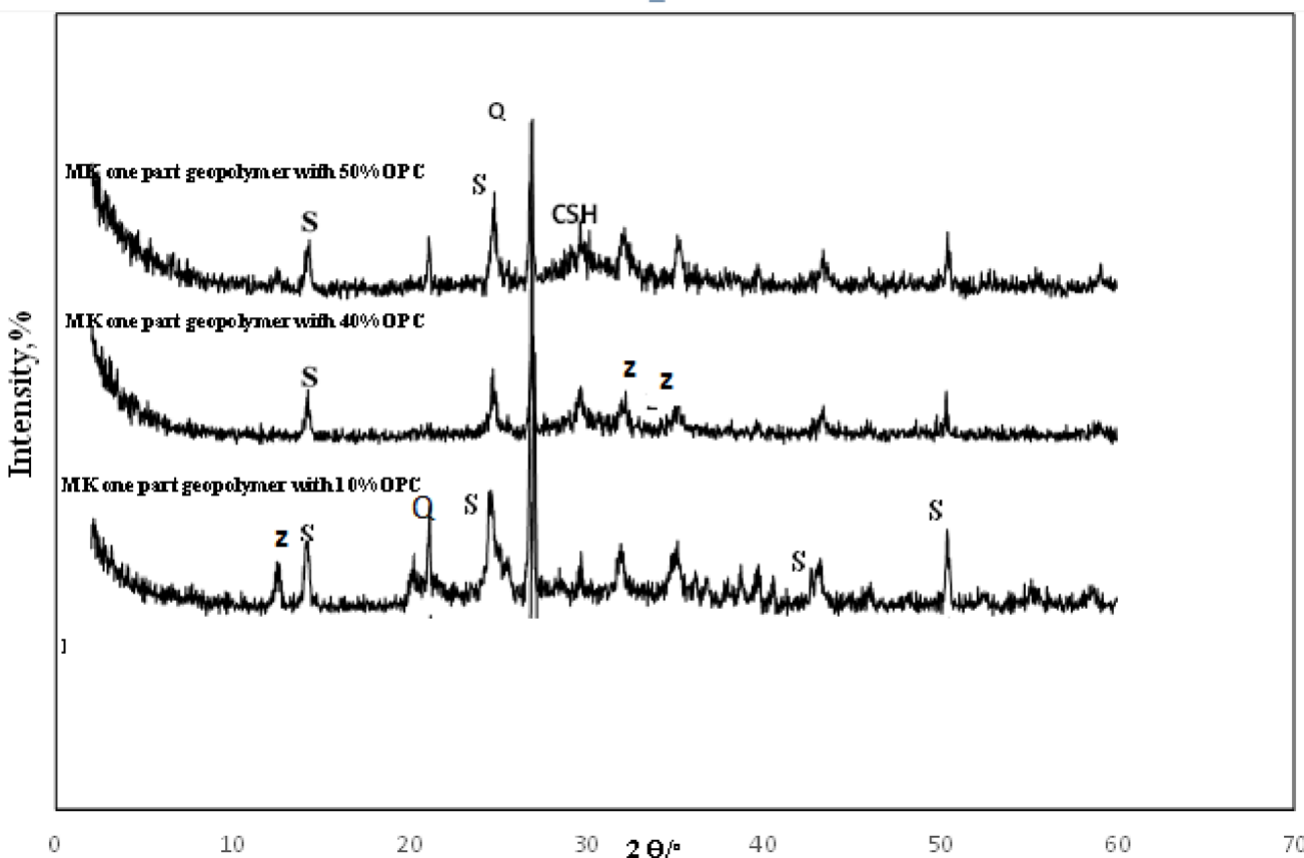
XRD pattern of 90 days one-part MK geopolymer pastes having various content of Portland cement [*Q* Quartz, *S* Sodalite, *Z* Zeolite, *CSH* calcium silicate hydrate].

The X-ray diffraction patterns (Figs. 5 and 6) show a broad hump in the 17–35° 2θ region, indicating a dominant amorphous phase^62–65^. Crystalline phases of zeolite, quartz (Q), sodalite, and (C, N)-A-S-H were detected in all samples. Additionally, calcium (aluminate) silicate hydrate (C-S-H) and calcite (C) were exclusively formed in high-cement-content mixtures (≥ 30% for normal geopolymer and ≥ 50% for the one-part sample).

Although both mixing methods produced similar combinations of amorphous and crystalline phases, the pre-dry mixing process yielded a greater amount of C-S-H. This is likely because the dry OPC had greater opportunity to react directly with the added water, facilitating more C-S-H formation than occurs in a pre-mixed alkaline activator solution (Fig. 6).

## Compressive strength measurements

**Fig. 7:**
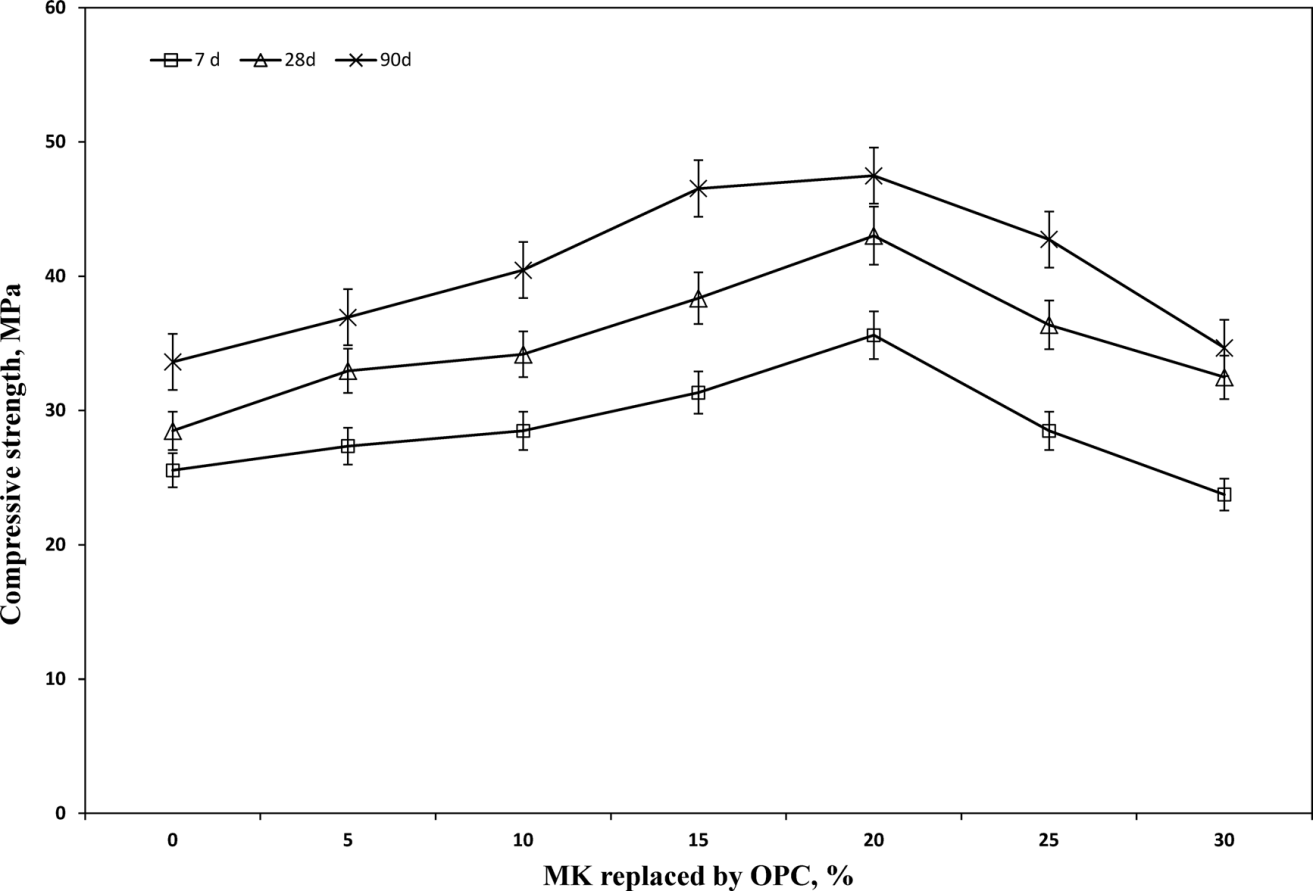
Compressive strength of metakaolin geopolymer having various ratio of Portland cement.

**Fig. 8:**
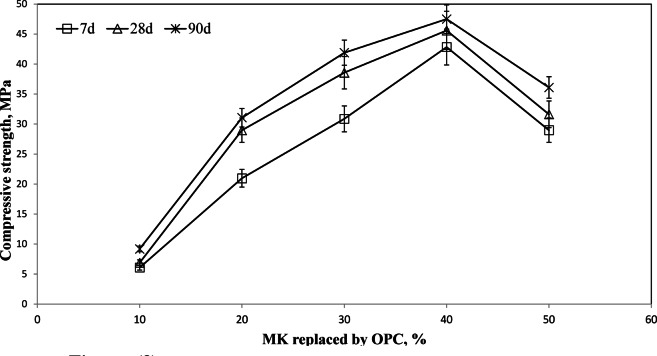
Compressive strength of one part metakaolin geopolymer pastes having various ratio of Portland cement.

The compressive strength of standard (two-part) and one-part self-cured geopolymer mixes was monitored for up to 90 days (Figs. 7 and 8). At 28 days, the one-part mixes demonstrated higher early strength (e.g.48 MPa) compared to the standard mixes (e.g., 45.3 MPa), despite incorporating a higher Portland cement content (up to 50% vs. 30%). This early strength gain is attributed to the internal heat released from hydration reactions that may partially enhance geopolymerization under ambient conditions. In contrast, the standard mixes may suffer from incomplete reactions and rapid heat loss from small specimens.

It can be seen from Table 3 that Ratio of Ca/Si indicats that beyond 20% CEM I there will a great susceptibility to the binding phases for transforming from the main amorphous constituent of geopolymer NASH to CASH which differ totally in their behavior and stability against sever environment.

However, the long-term (90-day) strength for both systems became comparable, indicating a similar final degree of geopolymerization^66,67^. The hardening mechanism is also temperature-dependent. At ambient temperature, it relies on a combination of nucleation from the liquid phase and geopolymer formation. At elevated temperatures, geopolymerization is the dominant mechanism. While heat accelerates early strength by increasing the reaction rate, slower curing at lower temperatures can produce a denser, tougher microstructure with superior long-term quality^68^.

### SEM investigatoions

**Fig. 9:**
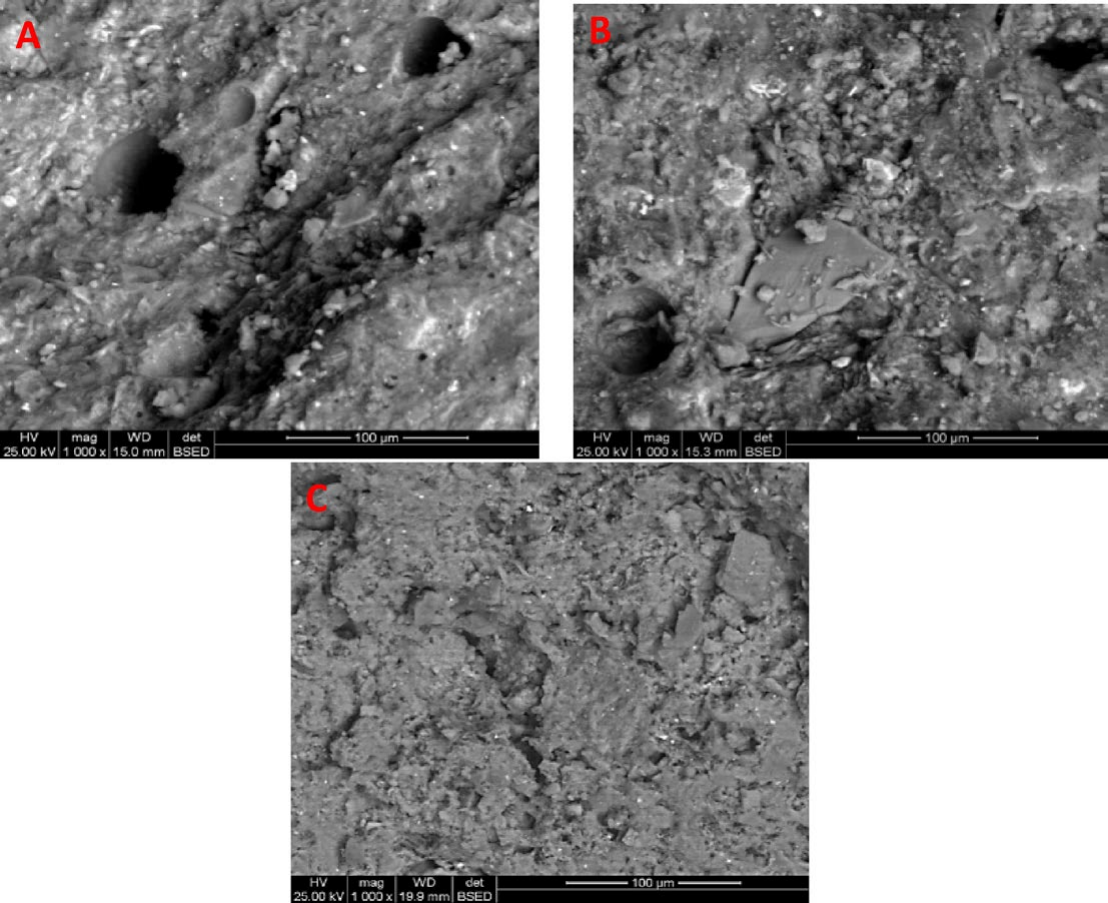
SEM of 90 days metakaolin geopolymer having various ratio of Portland cement; **a** control sample (0% replacement), **b** 20% replacement, **c** 30% replacement.

**Fig. 10:**
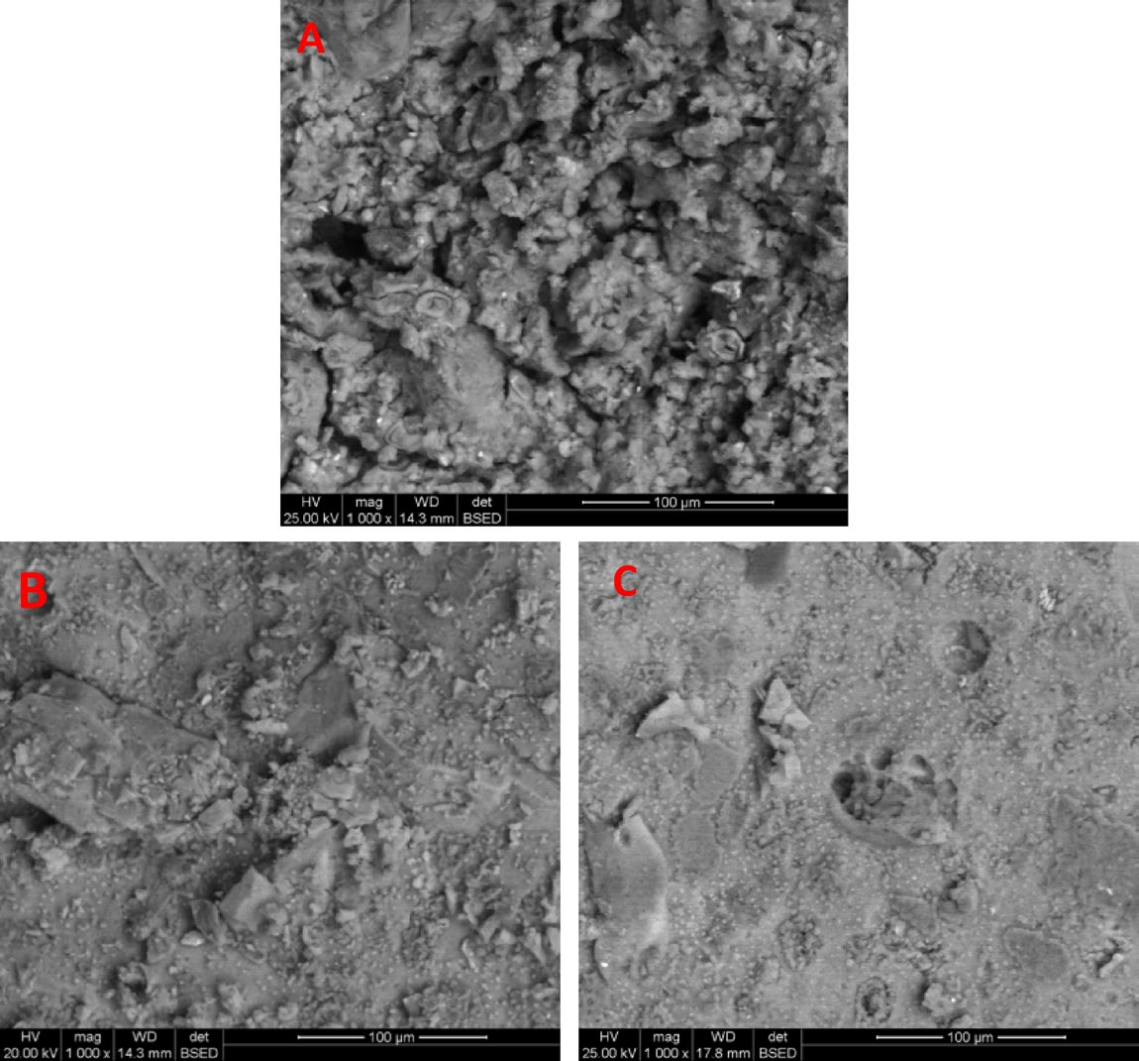
SEM of 90 days one-part metakaolin geopolymer pastes having various ratio of Portland cement: **a** 10% replacement, **b** 40% replacement, **c** 50% replacement.

Scanning electron micrographs of 90 days hardened normal geopolymer mixes incorporating various doses of porland cement up to 30% as represented in Fig. 9; as well as one-part geopolymer mixes prepared by inccoproating Portland cement up to 50% as represented in Fig. 10. We can observe from the microgpraph of the control paste that haven’t Portland cement (A) that it mainly composed of glassy phases of NASH that spread within the matrix, however there were many pores that can be seen through the micrograph which may results in the weakness of the structure. activated by NC, the formation of small geopolymer plates with the spreading of small micropores within the structure. However, using 20% Portland cement (B) results in the formation of massive geopolymer plates that mostly spread within the matrix as well as CSH phases that acts as nucleation sites for geopolymer formation^69–72^, and accumulation in addition their role in activating the reaction propagation through exothermic heat evoluted from hydration reaction leading to dense and compact structure^57,71^. Increasing the Portland cement content to 30% leads to the formation of microcracks as well as the decreased content of the formed amorphous geopolymer content as CASH and CSH predominate over NASH phases, which lead to the decrease in the homegenity.

Figure 10 shows scanning electron micrographs of 90-day-old, one-part geopolymer pastes with varying Portland cement (PC) content. At 10% PC (Image A), the structure is weak and microporous, featuring only small geopolymer plates. In contrast, the 40% PC sample (Image B) exhibits a dense, compact microstructure with massive, interconnected geopolymer plates. This enhanced structure is due to the heat from PC hydration, which boosts the formation of a cohesive binding phase of N–A–S–H and C–(A)–S–H gels. This observation is confirmed by the sample’s higher compressive strength, intense FTIR band, and disordered XRD phases. While the 50% PC sample (Image C) retains a cohesive structure, excessive hydration heat causes water evaporation and increased porosity.

## Conclusions

The main concluded remarks listed below:

- Preparation of self-cured geopolymer pastes by normal activation very as well as pre-activation one part method leads to the enhancement in the mechanical and microstructural properties of the prepared pastes.
- Partial replacement of metakaolin geopolymer by Portland cement up to 30% results in formation of amorphous powder and formation of dense structure and strength gain up to 20% followed by strength decrease.
- Partial replacement of pre activated one- part geopolymer by Portland cement up to 50% results in formation of amorphous powder and formation of dense structure and strength gain up to 40% followed by strength decrease.
- Compressive strength of hardened samples reflects the highest compressive strength for binder replaced by 20% Portland cement. It can be seen from the figures that the strength values for normal geopolymer mixes at 28 days were 40.4, 45.3. 34.2 MPa, however the strength values for 28 days for one- part system were 40.6, 48.0, 33.3 MPa.
- The results can offer low cost simple tool for preparation of self-cured binder using local materials as well Portland cement that can be applied without deleterious effects from activators up on mixing.
- The manuscript could make a solid contribution to the field of low-carbon binder development.

**Table 1:** Summary of representative studies on metakaolin- and fly ash–based geopolymer systems, including one-part solid activators, hybrid binders, and performance modifiers.

Duxson et al., 2007^33^	Metakaolin	NaOH + Sodium silicate (liquid)	Heat cured (60 °C)	0	High early strength; amorphous geopolymer gel	Classic two-part geopolymer system
Provis & van Deventer, 2009^34^	Fly ash	NaOH + Sodium silicate (liquid)	Ambient	0	High durability and good long-term strength	Fundamental study on geopolymer chemistry
Luukkonen et al., 2018^35^	Fly ash/slag	Anhydrous Na₂SiO₃ (solid)	Ambient	0	Comparable strength to liquid systems; easier handling	One-part geopolymer focus
Barboza-Chavez et al., 2020^36^	Fly ash + metakaolin + OPC clinker	NaOH + Sodium silicate	Room temp.	<30	Comparable to OPC; dense hydration matrix	Hybrid geopolymer–cement system
Bernal et al., 2021^37^	Fly ash + OPC	NaOH + Sodium silicate	Heat cured	20–40	Improved early strength; hybrid C–A–S–H + N–A–S–H gels	Hybrid geopolymer binder
Panda et al., 2020^38^	Metakaolin	Solid sodium silicate	Self-cured	0	Reduced water demand; moderate strength	Self-curing one-part geopolymer
Zhang et al., 2022^39^	Metakaolin	Alkali fusion method	Ambient	0	Enhanced reactivity; dense matrix	Alternative activation route
Barbosa et al., 2020^40^	Fly ash + OPC	NaOH + Sodium silicate	Ambient	10	Achieved 31 MPa at 28 days; no heat required	Hybrid cement for structural blocks
Al-Majidi et al., 2016^41^	Fly ash + OPC	NaOH + Sodium silicate	Self-cured	20	Achieved self-curing via internal heat and calcium	Early self-cured hybrid system
Luukkonen et al., 2020^42^	Metakaolin	Solid activator (Na₂SiO₃ + NaOH powder)	Ambient	0	Satisfactory strength and setting; user-friendly	Advancement in one-part design
Nath & Sarker, 2015^43^	Fly ash + GGBS	NaOH + Sodium silicate	Ambient	0	Enhanced early strength due to GGBS	Synergistic precursor combination
Tchakouté et al., 2016^44^	Metakaolin + OPC	NaOH + Sodium silicate	Ambient	10–30	Hybrid gel with increased strength	C–A–S–H and N–A–S–H coexistence
Aliabdo et al., 2019^45^	Metakaolin	NaOH + Sodium silicate	Ambient	0	High compressive strength and chemical resistance	Egyptian metakaolin study
Davidovits, 2015^46^	Various aluminosilicates	Alkaline activation	Various	0	Defined geopolymerization chemistry	Foundational reference
Current Study (2025)	Metakaolin + OPC	NaOH + Sodium silicate	Self-cured (ambient)	0–50	Target: self-curing + mechanical performance	Novel one-part self-cured hybrid system

**Table 2:** Chemical composition of starting materials. (Mass, %).

Oxide content (%)	SiO_2_	Al_2_O_3_	Fe_2_O_3_	CaO	MgO	SO_3_	K_2_O	Na_2_O	TiO_2_	MnO	*P*_2_O_5_	Cl-	L.O.I	Total
Kaolin	56.38	27. 61	1.32	0.18	0.06	0.06	0.04	0.08	3.73	-	0.13	0.05	10.17	99.76
MK (fired kaolin at 850 degree for 2 h)	57.50	35.10	1.59	0.64	0.17	0.25	0.15	0.12	2.85	0.00	0.13	0.06	1.14	99.70
CEM I	20.01	5.26	3.32	62.59	1.64	2.79	0.20	0.46	0.43	0.08	0.13	0.10	2.96	99.98

**Table 3:** Composition of the Geoplymer mixes.

Mix	MK, %	CEM I, %	NaOH, 10 M(ml)	Sodium silicate (ml)	Water/ binder	Total M_2_O/ Al_2_O_3_	SiO_2_/ Al_2_O_3_	Total M_2_O/ SiO_2_	Ca/Si
M1	100	0	33.50	66.50	0.396	0.100	2.310	0.030	0.019
M2	95	5	33.50	66.50	0.380	0.100	2.350	0.030	0.062
M3	90	10	33.50	66.50	0.380	0.110	2.400	0.030	0.125
M4	85	15	33.50	66.50	0.360	0.120	2.440	0.030	0.193
M5	**80**	**20**	**33.50**	**66.50**	**0.360**	**0.120**	**2.490**	**0.030**	**0.260**
M6	75	25	33.50	66.50	0.350	0.120	2.550	0.040	0.341
M7	70	30	33.50	66.50	0.350	0.130	2.610	0.040	0.422
N1	90	10	18.75	37.50	0.638	0.230	2.690	0.060	0.112
N2	80	20	18.75	37.50	0.576	0.260	2.820	0.060	0.234
N3	70	30	18.75	37.50	0.563	0.290	2.980	0.070	0.370
N4	**60**	**40**	18.75	37.50	**0.633**	**0.330**	**3.190**	**0.070**	**0.523**
N5	50	50	18.75	37.50	0.625	0.390	3.450	0.080	0.697

**Table 4:** Setting times of the Geoplymer mixes having various ratio of CEM I.

Mix	Initial time	Final time	W/C
0% (A)	6 h, 50 min	7 h, 10 min	0.396
5% (B)	1 h, 15 min	1 h, 35 min	0.38
10% (C)	45 min	65 min	0.38
15% (D)	27 min	45 min	0.36
20% (E)	20 min	32 min	0.35
25% (F)	12 min	25 min	0.35
30% (G)	8 min	20 min	0.35

## Data Availability

Data is provided within the manuscript.
